# Identification of a Set of Patient-Related Features to Foster Safe Prescribing of Specific Antipsychotics in the Elderly With Dementia

**DOI:** 10.3389/fpsyt.2020.604201

**Published:** 2020-10-30

**Authors:** João Pedro Aguiar, Catarina Bernardo, João Gama Marques, Hubert Leufkens, Filipa Alves da Costa

**Affiliations:** ^1^Faculdade de Farmácia, Research Institute for Medicines (iMED.ULisboa), Universidade de Lisboa, Lisbon, Portugal; ^2^Centro de Investigação Interdisciplinar Egas Moniz (CiiEM), Instituto Universitário Egas Moniz (IUEM), Caparica, Portugal; ^3^Hospital Júlio de Matos, Centro Hospitalar Psiquiátrico de Lisboa (CHPL), Lisbon, Portugal; ^4^Faculdade de Medicina, Clínica Universitária de Psiquiatria e Psicologia Médica, Universidade de Lisboa (FMUL), Lisbon, Portugal; ^5^Division of Pharmacoepidemiology and Clinical Pharmacology, Utrecht Institute for Pharmaceutical Sciences, Utrecht, Netherlands

**Keywords:** antipsychotics, health care research, prescription, patient safety, cognitive impairment

## Abstract

**Background:** Antipsychotics (APs) are widely used to manage behavioral and psychiatric symptoms in dementia, although with a variety of adverse drug reactions. Therefore, it is important to know which patient-related features should be considered to foster a safe prescribing of these medications.

**Objectives:** To compile and validate a set of patient-related features (PRFs) to foster safe prescribing of specific APs in the elderly with dementia; and to evaluate the feasibility of using them in clinical practice by analyzing the exhaustiveness of medical records.

**Method:** A rapid literature review was the starting point, where PRFs were identified through a search in PubMed combined with information from the Summary of Product Characteristics (SmPCs). In the next step, a two-round e-Delphi survey was undertaken, where a total of 450 participants were invited by e-mail, including prescribers and specialists in benefit-risk assessment. Finally, a cross-sectional study was undertaken, where 100 patients were randomly extracted from the psychiatric hospital database. Outcomes were defined as the assessment of the clinical relevance and feasibility of the PRFs, and the level of exhaustiveness of these features in medical records. Data analysis was performed using univariate statistics (IBM SPSS v.23.0).

**Results:** A total of 92 experts participated in the e-Delphi. Forty-seven PRFs obtained consensus, where 12 were applicable to haloperidol, 14 to olanzapine/risperidone, 13 to quetiapine, and 8 to aripiprazole. Age, comorbidities, and co-medications were rated as important features regardless of the prescribed drug. All PRFs were rated as always or frequently available and, if not, they were easy or partially easy to obtain. Age, comorbidities, and co-medications were always available in the medical records, whereas cognitive status (between 41.4 and 78.8%) or hepatic function (between 17.2 and 30.4%) presented a low-level of exhaustiveness.

**Conclusions:** Even though a high number of PRFs were rated as clinically relevant, some of them were identified as frequently missing from medical records. This may suggest that medical records should be complemented with other sources (e.g., nursing and pharmacy records) to ensure a safe prescribing of APs.

## Introduction

Antipsychotic (AP) medication is frequently used in several psychiatric conditions, including behavioral and psychiatric symptoms in dementia (BPSD), schizophrenia, and bipolar disorder (1). APs are commonly divided into two groups: typical and atypical. The first group has been on the market since 1950's and were associated with extrapyramidal symptoms (EPS). Over the years, atypical APs have been widely used compared to the typical group, given their lower risk of EPS (2, 3). However, they have been associated with metabolic syndrome and cardio- and cerebrovascular events (4–6).

Prescribing these medications to older individuals is common, particularly in nursing homes. The use of such medications in this age group is most of the times done off-label, as most of the evidence about their effectiveness and safety was extrapolated from younger adults. They can be used in the elderly to manage BPSD, which may affect up to 90% of the patients (1, 7). A systematic review found that risperidone, olanzapine, and aripiprazole showed greater efficacy than quetiapine, including the more severe cases. However, there is little evidence to suggest optimal duration of AP treatment, suggesting that a maximum of 6 weeks of treatment may be enough (8). Even though APs may have a greater benefit compared to non-pharmacologic measures, they also carry a greater risk (1).

Adverse drug reactions (ADRs) are common among the elderly, especially with psychotropic drugs (9). Multimorbidity, polypharmacy, and the use of inappropriate medications (PIMs – potentially inappropriate medications) are well-known risk factors associated with an increased risk of ADRs and, therefore, with higher costs, hospitalizations, and mortality (10, 11). In order to avoid ADRs, prescribing indicators have been identified and validated. Prescribing indicators are useful to: (a) optimize quality of healthcare delivery; (b) evaluate if medications are rationally used; (c) to audit and monitor practices in the context e.g., of quality circles, to describe and benchmark differences in practices; and (d) may also be used within clinical decision support systems (12–15). Different indicators may be divided according to different domains, such as safety (prescribing safety indicators) or quality (prescribing quality indicators). The first group has been defined as statements that describe prescribing events that may increase the risk of harm in patients (16). Prescribing safety indicators in mental health have been explored in a recent systematic review, where authors found that presence of PIMs, high risk medications, drug-disease interactions, and drug-related problems are examples of indicators that should be considered. They also found that 15.5% of those indicators were applicable to APs (17). A recent Delphi-study has developed prescribing safety indicators for medications used in mental health disorders, reporting 42 indicators considered to be of high or extreme risk for patient care. These included drug-disease and drug-drug interactions, inadequate monitoring, inappropriate dose, omissions, PIMs, and polypharmacy, most of which were applicable to Aps (18).

Even though some studies have been conducted to develop and validate prescribing safety indicators related to mental health, there is still the need to move from a population-based approach to patient-centered care. APs are a good example of a medication class with a wide range of receptor binding affinities, which may contribute to different ADR profile for each drug (19). Therefore, it may be important to know which patient-related features (PRFs) should be taken into account when prescribing specific APs to older patients with dementia. Therefore, our aims were to compile and validate a set of patient-features to foster safe prescribing of APs in older individuals with dementia and to evaluate the level exhaustiveness of such features in medical records of a mental health specialized hospital in Portugal.

## Materials and Methods

### Study Design

This study was divided into three steps: a rapid literature review to identify and compile possible PRFs, i.e., individual characteristics from the patients that may be used to foster safe prescribing of specific APs (e.g., quetiapine, olanzapine/risperidone, haloperidol, and aripiprazole) in the elderly with dementia; a consensus study to select the most clinically relevant PRFs for each drug; and a cross-sectional study where medical records from a Portuguese Psychiatry Hospital were reviewed to access their exhaustiveness regarding the features previously validated among comprehensively and validate PRFs.

### Compilation of Different PRFs Regarding AP Prescription for the Elderly With Dementia

Quetiapine, olanzapine/risperidone, haloperidol, and aripiprazole were chosen either based on their consumption pattern in older individuals with dementia or on their innovative mechanism of action, which may be an advantage on the risk-benefit ratio when prescribing the drug. A rapid literature review was performed using PubMed (20). Papers describing either PRFs used when prescribing APs or PRFs that should be monitored while using this medication in demented patients were included, considering the drug marketing authorizations of the selected APs. Summary of Product Characteristics (SmPCs) were used to supplement the results extracted from the rapid literature review. Age, renal and hepatic function, presence of comorbidities, and electrocardiogram results (EKG) were common features for all the selected drugs. PRFs specific for each drug were also extracted and summarized in Table 1.

**Table 1:** Drug-specific indicators extracted from the rapid literature review for the APs included in this study.

**Drug**	**Indicators specific for each of the selected drugs**
Haloperidol	Aged 65 or older Renal function Hepatic function Comorbidities EKG Concomitant medication Electrolyte disturbances (especially with potassium and magnesium)
Olanzapine/risperidone	Aged 65 or older Renal function Hepatic function Comorbidities EKG Hyperglycaemia/diabetes mellitus BMI > 30 kg/m^2^ Hypercholesterolemia High risk for metabolic syndrome High cardiovascular risk
Quetiapine	Aged 65 or older Renal function Hepatic function Comorbidities EKG Blood pressure High cardiovascular risk High risk for metabolic syndrome Hyperglycaemia/diabetes mellitus Hypercholesterolemia
Aripiprazole	Aged 65 or older Renal function Hepatic function EKG Sex Smoking habits High cardiovascular risk Hyperglycaemia/diabetes mellitus BMI > 30 kg/m^2^

*BMI, Body Mass Index; EKG, electrocardiogram*.

### Delphi Survey and Participants

To validate which PRFs would be more suitable to ensure a safe prescription of the previous selected APs in the elderly with dementia, a two-round Delphi survey were undertaken from July to September 2019. This method provides a systematic way to converge the expertise of individuals working in a specific area and gives guidance that is readily applicable to a particular context (21). A total of 450 participants were invited to participate in order to obtain a final sample of 100. Participants should be prescribers (which physicians and pharmacists from countries where this profession is allowed to prescribe) that may have a role in the management of elderly patients with dementia and experiencing BPSD or healthcare professionals specialized in the benefit-risk assessment. The panel size was a convenient sample number that was likely to yield stable results (21).

An initial sample of 38 features (7 for haloperidol, 10 for olanzapine/risperidone, 11 for quetiapine, and 10 for aripiprazole) were presented to the expert panel so they could rate them in terms of: (a) clinical relevance; (b) accessibility, i.e., how often they have access to the selected PRFs and, if needed, how easy it is to obtain them from elsewhere. Rating score were given according to a 5-item Likert scale: for clinical relevance assessment – 1 = Very important; 2 = Important; 3 = Equivocal; 4 = Less important; 5 = Not important; for how often do they have access – 1 = Always; 2 = Frequently; 3 = Sometimes; 4 = Rarely; 5 = Never; for how easy is to have them available – 1 = Very easy; 2 = Partially easy; 3 = Equivocal; 4 = Partially difficult; 5 = Difficult). The questionnaires were sent by e-mail and answered using a specific link generated by One Click Survey v. 19.08.91.

### Consensus Validation

When judging the clinical relevance, a mean score of 2 was used as the cut-off point to be agreed on and 75% as the consensus cut-off (22). In round one, scores ≤ 2 with a ≥ 75% consensus were automatically retained as important PRFs to be considered when prescribing APs for older individuals with dementia, whereas all others were included in round two together with new indicators suggested by the participants on the first round.

### Exhaustiveness of the PRFs in Medical Records of a Portuguese Psychiatric Hospital

The second part of this study was undertaken at a Portuguese Psychiatry Hospital – Hospital Júlio de Matos, Centro Hospitalar Psiquiátrico de Lisboa – between October and December of 2019. A sample of 100 patients were selected using a systematic method of choosing randomly the first patients of each month hospitalized in the psychogeriatric department between January of 2018 and December of 2019 who met the inclusion criteria (individuals aged 65 or older with dementia diagnosis and prescribed with APs). Data were extracted from medical records, which included sociodemographic information (age, sex, and education level), anthropometric measures (height, weight, and body mass index), clinical and laboratory data (comorbidities, medications, allergies, EKG, Minimal Mental Status – MMS, glycaemia, glycated hemoglobin – HbA1c, urea, creatinine, aspartate transaminase – AST, alanine aminotransferase – ALT, gamma-glutamyltransferase – gamma-GT, cholesterol, HDL, LDL triglycerides, sodium, potassium, and chloride), and drug-related data (co-medication, antipsychotic used, frequency, route of administration, and safety-related data – previous experience of ADRs).

### Data Analysis

Statistical analysis was performed using IBM SPSSv.26.0. Descriptive statistics were used for sociodemographic characterization of Delphi participants and to access responses obtained as well as to document the exhaustiveness of the PRFs in the medical records. Numerical variables were expressed using central tendency and dispersion measures (either as mean and standard deviations, whichever was applicable), and categorical variables as absolute and relative frequencies.

To assess the exhaustiveness of data entry, a specific classification was used based in a previous study: high (<1% missing values), medium (missing values between 1 and 15%), and low exhaustiveness (>15% missing values) (23). Anthropometric measures, EKG, MMS, and sociodemographic variables were considered present if described in medical records at the time of admission to the psychogeriatric department. For variables such as comorbidities, co-medications, and AP-related data, high-exhaustiveness was considered if those variables were available in the last update of the medical record. Laboratory values and biomarkers assessment (e.g., blood pressure) were searched for a 6-months period prior to the index date (i.e., date of last medical record update during the study period) and were considered to present high-exhaustiveness if they had at least 3 measurements. For indicators that may result in a final score (e.g., cardio and cerebrovascular risk, frailty/risk of falls) were classified based on the exhaustiveness of the individual data needed to calculate them.

## Results

### Consensus Results

#### Participants' Characteristics

From the initial 126 participants who agree to participate, there were three dropouts from the study and 31 incomplete questionnaires which were excluded. A total of 92 participants were retained, where 53.3% (*n* = 49) were male and 39.1% (*n* = 36) belonged to the age group of 30–39 years old. Almost half of the sample (43.5%; *n* = 40) had a PhD degree and had <10 years of working experience (43.4%; *n* = 40). The majority of participants were either psychiatrists (25.0%; *n* = 23) or internal medicine physicians (25.0; *n* = 23), followed by pharmacists able to prescribe (15.2%; *n* = 14), pharmacologists (10.0%; *n* = 9), gerontologists (9.8%; *n* = 7), general practitioners (6.5%; *n* = 6), epidemiologists (5.4%; *n* = 5), cardiologists (2.2%; *n* = 2), neurologists (2.2%; *n* = 2), and palliative care physicians (1.1%; *n* = 1). Table 2 summarizes the sociodemographic characterization of the panel experts.

**Table 2:** Sociodemographic characteristics of the Delphi survey participants.

**Sociodemographic characteristics**	***N* = 92**
**Sex**, ***n*** **(%)**	
Male	49 (53.3)
Female	43 (46.7)
**Age**, ***n*** **(%)**	
20–29	14 (15.2)
30–39	36 (39.1)
40–49	15 (16.3)
50–59	13 (14.1)
≥ 60	14 (15.2)
**Educational degree**, ***n*** **(%)**	
Bachelor	15 (16.3)
Master	32 (34.8)
PhD	40 (43.5)
Other	5 (5.4)
**Speciality/area of expertise**, ***n*** **(%)**	
Psychiatry	23 (25.0)
Internal medicine	23 (25.0)
Clinical pharmacy	14 (15.2)
Pharmacology	9 (10.0)
Gerontology	7 (9.8)
General practice	6 (6.5)
Epidemiology	5 (5.4)
Cardiology	2 (2.2)
Neurology	2 (2.2)
Palliative care	1 (1.1)

#### PRFs Selected in the Two-Round Delphi Survey

A total of 61 PRFs (13 for haloperidol, 18 for olanzapine/risperidone, 21 for quetiapine, and 20 for aripiprazole) were presented to the expert panel, where 38 were retrieved from the literature and 23 were suggested by the participants after the first round. In the end of the second round, 47 PRFs were retained, where 12 (25.5%) were selected for haloperidol, 14 (29.8%) for olanzapine/risperidone, 13 (27.7%) for quetiapine, and 8 (17.0%) for aripiprazole.

Age, comorbidities, and co-medications were rated as important features for safe prescribing of antipsychotics in the elderly and were found in all the selected drugs. Table 3 summarizes the clinical relevance, availability of specific PRFs in medical records or possibility for obtaining them when not available. All the selected features were either always or frequently available in daily practice and, if not, all of them were easy or partially easy to request.

**Table 3:** PRFs selected through the Delphi survey for each drug as the most important ones to foster safe prescribing of APs in older individuals.

**Indicators**	**Panel survey score (mean** **±** **SD)**		
	**Clinical relevance^†^**	Feasibility^*^	**Availability when asked^‡^**
**Haloperidol**			
Age	1.05 ± 0.7	1.25 ± 0.76	1.10 ± 0,39
Hepatic function	2.00 ± 0.9	1.73 ± 0.80	1.81 ± 0.72
Comorbidities	1.50 ± 0.6	1.48 ± 0.59	1.73 ± 0.54
EKG	1.60 ± 0.6	1.94 ± 0.77	2.16 ± 0.76
Electrolyte disturbances	1.90 ± 0.9	1.20 ± 0.86	1.91 ± 0.74
Co-medications	1.30 ± 0.7	1.41 ± 0.81	1.78 ± 0.76
Labeled indication	1.50 ± 0.7	1.27 ± 0.54	1.67 ± 0.87
Frailty/risk of falls	1.60 ± 0.7	1.77 ± 0.76	1.91 ± 0.85
Previous ADRs	1.40 ± 0.5	1.92 ± 0.78	2.24 ± 1.06
Cognitive status	1.80 ± 0.8	1.66 ± 0.57	1.97 ± 0.78
Benefit-risk ratio assessment	1.30 ± 0.5	1.51 ± 0.64	1.92 ± 0.89
Presence of Parkinson Disease	2.00 ± 0.80	1.89 ± 0.77	1.75 ± 0.74
**Olanzapine/risperidone**			
Age	1.03 ± 0.24	1.03 ± 0.24	1.06 ± 0.33
Renal function	1.78 ± 0.58	1.78 ± 0.58	1.63 ± 0.69
Hepatic function	1.90 ± 0.67	1.90 ± 0.97	1.67 ± 0.67
Comorbidities	1.40 ± 0.54	1.40 ± 0.54	1.63 ± 0.51
Co-medications	1.50 ± 0.79	1.50 ± 0.79	1.70 ± 0.51
Hyperglycaemia/diabetes mellitus	1.76 ± 0.55	1.76 ± 0.55	1.59 ± 0.54
Weight	2.16 ± 0.95	2.16 ± 0.95	1.80 ± 0.65
Cardiovascular risk	2.03 ± 0.68	2.03 ± 0.68	1.97 ± 0.72
Labeled indication	1.56 ± 0.87	1.56 ± 0.87	1.58 ± 0.76
Frailty/Risk of falls	1.82 ± 0.91	1.82 ± 0.91	1.84 ± 0.82
Previous ADRs	1.94 ± 0.94	1.94 ± 0.94	2.06 ± 0.77
Cognitive status	1.71 ± 0.80	1.71 ± 0.80	1.63 ± 0.66
Benefit-risk ratio assessment	1.64 ± 0.77	1.64 ± 0.77	1.68 ± 0.77
Cerebrovascular risk	1.93 ± 0.48	1.93 ± 0.88	1.87 ± 0.89
**Quetiapine**			
Age	1.60 ± 0.80	1.15 ± 0.35	1.06 ± 0.33
Hepatic function	1.90 ± 0.80	1.69 ± 0.68	1.64 ± 0.61
Comorbidities	1.50 ± 0.60	1.41 ± 0.63	1.49 ± 0.50
EKG	2.00 ± 0.70	2.10 ± 0.74	1.77 ± 0.62
Co-medication	1.40 ± 0.50	1.44 ± 0.70	1.64 ± 0.51
Cardiovascular risk	1.90 ± 0.70	2.20 ± 1.00	1.94 ± 0.68
Blood pressure	2.00 ± 0.80	1.46 ± 0.60	1.25 ± 0.61
Labeled indication	1.50 ± 0.60	1.49 ± 0.61	1.59 ± 0.81
Frailty/risk of falls	1.60 ± 0.70	1.79 ± 0.75	1.71 ± 0.74
Previous ADRs	1.60 ± 0.80	1.82 ± 0.72	1.92 ± 0.81
Cognitive status	1.70 ± 0.80	1.65 ± 0.61	1.68 ± 0.74
Benefit-risk ratio assessment	1.50 ± 0.70	1.59 ± 0.63	1.67 ± 0.81
Cerebrovascular risk	1.80 ± 0.80	1.89 ± 0.82	1.93 ± 0.92
**Aripiprazole**			
Age	1.60 ± 0.80	1.12 ± 0.31	1.03 ± 0.24
Comorbidities	1.60 ± 0.80	1.49 ± 0.55	1.66 ± 0.57
Co-medications	1.60 ± 0.70	1.69 ± 0.95	1.69 ± 0.95
Cardiovascular risk	2.00 ± 0.80	2.20 ± 1.00	1.99 ± 0.79
Labeled indication	1.60 ± 0.70	1.77 ± 1.03	1.77 ± 0.98
Benefit-risk ratio assessment	1.50 ± 0.70	1.71 ± 1.01	1.96 ± 0.98
Cerebrovascular risk	1.90 ± 0.70	2.01 ± 0.99	1.82 ± 0.93
Clinical response and tolerability to previous APs	1.60 ± 0.70	2.04 ± 0.99	2.16 ± 0.91

*ADRs, Adverse Drug Reactions; Aps, Antipsychotics; EKG, Electrocardiogram*.

† *Rating scale: 1-Very important; 2-Important; 3-Equivocal; 4-Less important; 5-Not important*.

* *Feasibility in clinical practice means how often do healthcare professionals, namely prescribers, have access to the selected indicators in their daily practice; rating scale: 1-Always; 2-Frequently; 3-Sometimes; 4-Rarely; 5-Never*.

‡ *Rating scale: 1-Very easy; 2-Partially easy; 3-Equivocal; 4-Partially difficult; 5-Difficult*.

### Exhaustiveness of PRFs in Medical Records

Age, comorbidities, co-medications, and the indication for which the drug was being used presented a high-level exhaustiveness in the medical records independently of the drug used. For haloperidol, electrolyte disturbances and the presence of Parkinson disease were also extensively described in the charts, whereas for olanzapine/risperidone the same result was found for the presence of diabetes. Conversely, hepatic function (haloperidol – 22.2%; olanzapine – 30.4%; risperidone – 17.2%; quetiapine – 30.3%), EKG (haloperidol – 44.4%; quetiapine – 42.4%), cognitive status (haloperidol – 66.7%; olanzapine – 60.9%; risperidone – 41.4%; quetiapine – 78.8%), and weight (olanzapine – 100.0%; risperidone – 100.0%) presented a low-level of exhaustiveness. Renal function (olanzapine – 12.5%; risperidone – 6.9%) and blood pressure (12.1%) presented a medium-level of exhaustiveness. Table 4 summarizes all the results described.

**Table 4:** Exhaustiveness of PRFs selected through the Delphi survey in medical records of older individuals with dementia.

**Indicators**	**Exhaustiveness of medical records**		
	***n***	**%**	Description^*^
**Haloperidol (*****n*** **=** **9)**			
Age	0	0.0	High
Hepatic function	2	22.2	Low
Comorbidities	0	0.0	High
EKG	4	44.4	Low
Electrolyte disturbances	0	0.0	High
Co-medications	0	0.0	High
Labeled indication	0	0.0	High
Frailty/risk of falls	n/a	n/a	n/a
Previous ADRs	n/a	n/a	n/a
Cognitive status	6	66.7	Low
Benefit-risk ratio assessment	n/a	n/a	n/a
Presence of Parkinson Disease	0	0.0	High
**Olanzapine (*****n*** **=** **23)**			
Age	0	0.0	High
Renal function	3	12.5	Medium
Hepatic function	7	30.4	Low
Comorbidities	0	0.0	High
Co-medications	0	0.0	High
Presence of hyperglycaemia	6	26.1	Low
Presence of diabetes mellitus	0	0.0	High
Weight	23	100.0	Low
Cardiovascular risk	n/a	n/a	n/a
Labeled indication	0	0.0	High
Frailty/Risk of falls	n/a	n/a	n/a
Previous ADRs	n/a	n/a	n/a
Cognitive status	14	60.9	Low
Benefit-risk ratio assessment	n/a	n/a	n/a
Cerebrovascular risk	n/a	n/a	n/a
**Risperidone (*****n*** **=** **29)**			
Age	0	0.0	High
Renal function	2	6.9	Medium
Hepatic function	5	17.2	Low
Comorbidities	0	0.0	High
Co-medications	0	0.0	High
Presence of hyperglycaemia	3	10.3	Medium
Presence of diabetes mellitus	0	0.0	High
Weight	29	100.0	Low
Cardiovascular risk	n/a	n/a	n/a
Labeled indication	0	0.0	High
Frailty/risk of falls	n/a	n/a	n/a
Previous ADRs	n/a	n/a	n/a
Cognitive status	12	41.4	Low
Benefit-risk ratio assessment	n/a	n/a	n/a
Cerebrovascular risk	n/a	n/a	n/a
**Quetiapine (*****n*** **=** **33)**			
Age	0	0.0	High
Hepatic function	10	30.3	Low
Comorbidities	0	0.0	High
EKG	14	42.4	Low
Co-medication	0	0.0	High
Cardiovascular risk	n/a	n/a	n/a
Blood pressure	4	12.1	Medium
Labeled indication	0	0.0	High
Frailty/risk of falls	n/a	n/a	n/a
Previous ADRs	n/a	n/a	n/a
Cognitive status	26	78.8	Low
Benefit-risk ratio assessment	n/a	n/a	n/a
Cerebrovascular risk	n/a	n/a	n/a
**Aripiprazole (*****n*** **=** **6)**			
Age	0	0.0	High
Comorbidities	0	0.0	High
Co-medications	0	0.0	High
Cardiovascular risk	n/a	n/a	n/a
Labeled indication	0	0.0	High
Benefit-risk ratio assessment	n/a	n/a	n/a
Cerebrovascular risk	n/a	n/a	n/a
Clinical response and tolerability to previous APs	n/a	n/a	n/a

*EKG, electrocardiogram; n/a, not available*.

* *High exhaustiveness: <1% missing values; medium exhaustiveness: between 1 and 15%; low exhaustiveness: >15% missing values)*.

## Discussion

### Main Findings

In this study, we found that 47 of the initial 61 PRFs were retained as relevant in clinical practice to safely prescribe an AP to an older individual with dementia. Of those indicators, most of them were specific for the selected drugs, and participants reported that all of them were always or frequently available in the medical records. If not available, all of them were easy or partially easy to request. When evaluating their exhaustiveness in the medical records, we found that age, comorbidities, and co-medications were always available, whereas cognitive status or hepatic function presented a low-level of exhaustiveness.

To ensure safe prescribing of APs in the elderly, it is important to consider not only drug-related issues, but also patient-related features. As most other psychotropic drugs, APs are known to have different mechanisms of action, given their binding affinity to specific receptors, which may lead to different ADRs. For instance, haloperidol is known to cause QT-prolongation or parkinsonism, whereas olanzapine and risperidone are known to be associated with metabolic syndrome (24, 25). For this reason, data on EKG, glycemia, cholesterol, and other laboratory values should be available in order not only to monitor patients already instituted therapy, but also to make sure that the AP being prescribed for the first time will not increase the risk of ADRs. We found that EKG was an important feature when prescribing haloperidol or quetiapine, even though a low-level of exhaustiveness was obtained, albeit reported as easy to request. Similar results were found for olanzapine and risperidone when evaluating the presence of hyperglycaemia, hypercholesterolemia, and weight. This indicates that prescribers know what is important to consider when prescribing these drugs, but data may not be fully available given the organization of the healthcare system. In Portugal, data from in- and out-patient settings are not always integrated, which leads to a different level of exhaustiveness when both settings are compared. Most importantly, this gap makes the data available different for each prescriber, i.e., a general practitioner may have different access to a certain type of indicators in comparison with a psychiatrist.

Another indicator rated as important was cognitive status, which was absent in most medical records. It is known that in patients with cognitive impairment, like demented patients, the assessment of cognitive status is important when prescribing APs (26). These safety issues are crucial when prescribing medications to older adults, especially in patients with psychiatric symptoms where multiple medications may interact with each other, resulting in exacerbation of cognitive impairment.

Even though frailty status, and cardio- and cerebrovascular risk were not available in the medical records, these scores were rated as clinically relevant when prescribing APs to elderly patients with dementia. It is known that these drugs may be associated with an increased risk of cardio- and cerebrovascular events (4–6). These scores sometimes are not available directly in the medical record of the hospital, but nowadays many online calculators are available. Therefore, if the data needed to calculate the scores is available in the medical records, prescribers may be able to calculate them and take these risks into account when prescribing, especially atypical APs (e.g., olanzapine, risperidone, and quetiapine). Another important aspect is the fact that some PRFs may be available in the nursing records (e.g., blood pressure, weight), which may be missed if prescribers do not look for it when prescribing atypical APs, where the risk of developing metabolic syndrome is high. So, it is important to acknowledge the contribution of different patient information sources to ensure a safe prescribing of such medications (27).

Few studies have evaluated the need to validate prescribing quality and safety indicators, i.e., indicators for evaluating the quality of prescribing (e.g., adherence, presence of polypharmacy), and also the safety when prescribing to older individuals (e.g., presence of drug-drug interactions, concurrent use of more than one AP). One of the aims of developing a set of such indicators was to prevent/minimize the occurrence of ADRs. However, such indicators are mostly population-oriented, and do not consider the need to look for specific features that may be important when prescribing a specific AP, e.g., relevant for haloperidol, but not so important, for instance, for olanzapine. As some of these elder patients may be on more than one AP, a combination of features may need to be accessed prior to a prescription. Moreover, knowing that this population is highly heterogeneous, there may be some patients where specific features may be more important than in others. For instance, in patients with previous history of cardiac arrhythmias, an EKG for evaluating the QT segment may be needed ahead of the prescription, so that prescribers may select among APs that do not increase the risk of heart block.

### Impact on Practice

To the authors' best knowledge, this is one of the few studies validating patient-related features that may contribute to a safer prescribing pattern of specific APs, like haloperidol, olanzapine, risperidone, quetiapine, and aripiprazole. Current prescribing culture is more focused on effectiveness rather than safety, which in older patients with dementia may increase their odds of experiencing ADRs. Even though prescribers have identified a set of patient-related features with clinical relevance, a low-level of exhaustiveness in our country was found which may be a reality in other countries with a similar healthcare system. This may show the current need not only to integrate the different healthcare software, but also to unify the entire healthcare setting in order to optimize patient medications, especially psychotropic drugs. Future work will include the development of an algorithm to be integrated in a digital tool or app, that may be able to include all these important variables in order to ensure safe prescribing of APs in this population group.

## Limitations

Some limitations have been identified and are worth acknowledging. First, selection bias may be present in both samples (expert panel and in-hospital patients). However, we believe that in the sample retrieved from the hospital this bias may be reduced, given that we used a quasi-random methodology and patients were extracted from a specialized hospital in psychiatric illness, which may contribute to a more homogeneous distribution of patients' characteristics. Secondly, misclassification bias may be present given that most information was retrieved from medical charts of different physicians. We believe that this bias was minimized given that the authors have coded the variables according to a pre-defined dataset, which may have contributed to a more homogeneous coding system. Finally, this data may not be generalized for a larger population.

## Conclusions

To conclude, this study has validated a set of patient-related features, like age, comorbidities, co-medications, renal and hepatic function, and cognitive status as relevant items to consider in daily practice when prescribing specific APs to older individuals with dementia. All of them always or frequently were available in medical records and, when absent, considered easy to request. However, a low-level of exhaustiveness was found in medical records for certain features, such as cognitive status, hepatic function, and weight. Future work will focus on the development of drug-specific algorithms to be included in a digital platform or app to foster safe prescribing of such medications in older individuals with dementia.

## Data Availability Statement

The original contributions presented in the study are included in the article/supplementary material, further inquiries can be directed to the corresponding author/s.

## Ethics Statement

The studies involving human participants were reviewed and approved by the Ethics Committee of Egas Moniz (658/2018) and the Ethics Committee of Centro Hospitalar Psiquiátrico de Lisboa (0019/2019). Written informed consent for participation was not required for this study in accordance with the national legislation and the institutional requirements.

## Author Contributions

FA, HL, and JA conceived and designed the study. CB, JA, and JG collected, analyzed, and interpreted the data. JA prepared the manuscript. All the authors have critically reviewed the manuscript until its final version.

## Conflict of Interest

The authors declare that the research was conducted in the absence of any commercial or financial relationships that could be construed as a potential conflict of interest.
